# Correlation between amide proton transfer-related signal intensity and diffusion and perfusion magnetic resonance imaging parameters in high-grade glioma

**DOI:** 10.1038/s41598-021-90841-z

**Published:** 2021-05-27

**Authors:** Masanori Nakajo, Manisha Bohara, Kiyohisa Kamimura, Nayuta Higa, Takashi Yoshiura

**Affiliations:** 1 Department of Radiology, Kagoshima University Graduate School of Medical and Dental Sciences, 8-35-1 Sakuragaoka, Kagoshima, 890-8544 Japan; 2 Department of Neurosurgery, Kagoshima University Graduate School of Medical and Dental Sciences, 8-35-1 Sakuragaoka, Kagoshima, 890-8544 Japan

**Keywords:** Medical research, Cancer in the nervous system, CNS cancer

## Abstract

Amide proton transfer (APT) imaging is a magnetic resonance (MR) molecular imaging technique that is sensitive to mobile proteins and peptides in living tissue. Studies have shown that APT-related signal intensity (APTSI) parallels with the malignancy grade of gliomas, allowing the preoperative assessment of tumor grades. An increased APTSI in malignant gliomas has been attributed to cytosolic proteins and peptides in proliferating tumor cells; however, the exact underlying mechanism is poorly understood. To get an insight into the mechanism of high APTSI in malignant gliomas, we investigated the correlations between APTSI and several MR imaging parameters including apparent diffusion coefficient (ADC), relative cerebral blood volume and pharmacokinetic parameters obtained in the same regions-of-interest in 22 high-grade gliomas. We found a significant positive correlation between APTSI and ADC (ρ = 0.625 and 0.490 for observers 1 and 2, respectively; p < 0.001 for both), which is known to be inversely correlated with cell density. Multiple regression analysis revealed that ADC was significantly associated with APTSI (p < 0.001 for both observers). Our results suggest possible roles of extracellular proteins and peptides in high APTSI in malignant gliomas.

## Introduction

Chemical exchange saturation transfer (CEST) is a novel imaging contrast mechanism in magnetic resonance imaging (MRI)^1^. CEST is based on the physical exchange of protons between solutes and water molecules, which is called chemical exchange. Amide proton transfer (APT) is a specific type of CEST for amide protons in mobile proteins and peptides^2^. APT imaging is considered the most clinically feasible CEST imaging because its specific resonance frequency is remote (3.5 ppm down field) from the water resonance frequency. Studies have shown the potential clinical uses of APT imaging, especially in assessing brain tumors. In diffuse gliomas, several studies have shown that APT-related signal intensity (APTSI) increases with increasing malignancy grades as defined by the World Health Organization (WHO)^3–6^. Moreover, APTSI has been reported as a useful imaging marker in distinguishing recurrent gliomas after chemoradiation therapy from radiation necrosis^7–9^, evaluating early response to treatment^10^, and predicting prognosis^11^. The most widely accepted hypothesis for higher APTSI in malignant gliomas is that APTSI reflects cytosolic protein that would increase in malignant gliomas with a higher proliferative activity. Nonetheless, extracellular proteins and peptides such as those in fluid-filled cysts and hemorrhage can be sources of APTSI^12,13^. Thus, the underlying mechanism of high APTSI in malignant gliomas is not fully understood. One possible approach to get insight into this mechanism is to investigate the correlations between APTSI and other imaging parameters. This study correlates APTSI with diffusion and perfusion MRI parameters in malignant gliomas.

## Materials and methods

### Patients

This retrospective study was approved by the Institutional Review Board (IRB) of Kagoshima University Hospital (approval number: 190103) and the need to obtain informed consent from the patients was explicitly waived by the IRB which approved this study’s protocol. The study was performed in accordance with the Declaration of Helsinki. From August 2016 to January 2018, APT-weighted imaging (APTWI) was performed as a part of the preoperative MRI examination protocol for brain tumors in our hospital. In this study, the inclusion criteria were (1) MRI examination including APTWI, diffusion-weighted imaging (DWI), dynamic contrast-enhanced (DCE) imaging, and dynamic susceptibility contrast (DSC) perfusion imaging on the same day within 14 days before surgery and (2) pathological confirmation of high-grade diffuse glioma (either grade III or IV). Statuses of the isocitrate hydrogenase (IDH) mutation and 1p/19q codeletion were determined for each tumor using the Sanger sequencing and fluorescence in situ hybridization, respectively. The following patients were excluded from the study: (1) patients who received any treatment before MRI examination, (2) those with recurrent tumor, and (3) those with severe artifacts that preclude appropriate image analysis.

### MRI

All patients underwent MRI examination using a 3.0 T imager (Ingenia, Philips, Best, The Netherlands) and a 15-channel head coil. APTWI was performed using a two-channel parallel transmission scheme with a saturation pulse with a duration of 2 s (40 × 50 ms, sinc-Gauss-shaped elements) and a saturation power level of B1 (rms = 2.0 μT)^14,15^ at 25 saturation frequency offsets ranging from − 6 to + 6 ppm with a step of 0.5 ppm and one far-off-resonant frequency (− 1560 ppm) for signal normalization. The other imaging parameters were as follows: fast spin-echo readout with driven equilibrium refocusing; echo train length (ETL) = 128; sensitivity encoding (SENSE) acceleration factor = 1; repetition time (TR) = 3600 ms; echo time (TE) = 4.8 ms; matrix = 128 × 128 (reconstructed to 256 × 256); slice thickness = 5 mm, field of view (FOV) = 230 × 230 mm; and scan time = 2 min and 20 s for one Z-spectrum. A ΔB0 map for off-resonance correction was acquired separately using a 2D gradient-echo with identical spatial resolution for a point-by-point ΔB0 correction^14,15^.

DWI was performed using a single-shot spin-echo echo-planar imaging (EPI) sequence using the following imaging parameters: b value = 0 and 1000 s/mm^2^; number of diffusion-encoding direction = 3; SENSE acceleration factor = 2.5 (phase); TR = 4250 ms; TE = 63 ms; flip angle (FA) = 90°; number of signals averaged = 2; matrix = 128 × 128 (reconstructed to 256 × 256); number of slices = 24; slice thickness = 5 mm; interslice gap = 1 mm; FOV = 230 × 230 mm; and scan time = 1 min and 40 s.

DCE imaging was performed using a 3D fast field-echo sequence using the following imaging parameters: SENSE acceleration factor = 1.8 (phase) × 1.1 (slice); TR = 6.1 ms; TE = 4.6 ms; FA = 15°; matrix = 128 × 128 (reconstructed to 256 × 256); number of slices = 9; slice thickness = 6 mm; FOV = 230 × 230 mm; and scan time = 5 min and 19 s. Ninety-five dynamic images were obtained at an interval of 3.4 s starting at 30 s before the contrast injection. A power injector (Sonic Shot 7, Nemoto Kyorindo, Tokyo, Japan) was used for intravenous injection of a Gadolinium-based contrast agent (GBCA) (either meglumine gadopentetate or meglumine gadoterate, 0.05 mmol/kg) at a rate of 2 ml/s followed by a saline flush (20 ml, 2 ml/s). A pre-contrast 3D fast filed-echo imaging was performed before the DCE imaging to obtain a pre-contrast T1 value of each voxel using the same imaging parameters as those in the DCE scan, except for an FA of 5°.

DSC perfusion imaging was performed immediately after the completion of DCE imaging using a single-shot field-echo EPI sequence using the following imaging parameters: SENSE acceleration factor = 2.3 (phase); TR = 1610 ms; TE = 40 ms; FA = 75°; matrix = 112 × 112 (reconstructed to 224 × 224); number of slices = 24; slice thickness = 5 mm; interslice gap = 1 mm; FOV = 230 × 230 mm; scan time = 1 min and 41 s. Dynamic imaging was started 8 s before contrast injection. A GBCA (0.05 mmol/kg) was injected at a rate of 4 ml/s followed by a saline flush (20 ml, 4 ml/s).

Post-contrast transverse T1-weighted spin-echo images (T1WIs) were obtained using the following imaging parameters: TR = 410 ms; TE = 10 ms; number of signals averaged = 1; matrix = 304 × 304 (reconstructed to 512 × 512); number of slices = 24; slice thickness = 5 mm; interslice gap = 1 mm; FOV = 230 × 230 mm; and scan time = 2 min and 46 s.

Other standard MRI sequences included pre-contrast T1WI, T2-weighted imaging, fluid-attenuated inversion recovery imaging, susceptibility-weighted imaging, and post-contrast 3D time-of-flight MR angiography; these sequences were not actively used in this study, although pre-contrast T1WIs were used to confirm contrast enhancement.

### Generation of parametric maps

The B0 inhomogeneity correction for APT image data was performed using a separately obtained B0 map as previously described. Subsequently, APTWIs were generated using pixel-by-pixel mapping of APTSI, which is defined as the magnetization transfer ratio asymmetry (MTRasym) at 3.5 ppm: MTRasym (3.5 ppm)

$$\begin{aligned} {\text{APTSI }} & = {\text{ MTRasym }}\left( {{3}.{\text{5 ppm}}} \right) \\ & = \, \left[ {{\text{S }}\left( { - {3}.{\text{5 ppm}}} \right){-}{\text{S }}\left( { + {3}.{\text{5 ppm}}} \right)} \right]/{\text{S}}0, \\ \end{aligned}$$

where S (− 3.5 ppm), S (+ 3.5 ppm), and S0 are the signal intensities obtained at − 3.5 ppm, + 3.5 ppm, and − 1560 ppm, respectively. The APT image analyses were performed using an in-house plug-in software running on ImageJ (v1.47v; National Institutes of Health, Maryland, USA).

Apparent diffusion coefficient (ADC) maps were obtained from the DWI at b values of 0 and 1000 s/mm^2^ assuming the mono-exponential signal decay.

Analysis of the DCE images was performed using the extended Tofts model on an image analysis workstation (IntelliSpace Portal, Philips, The Netherlands). The maps of the following four pharmacokinetic parameters were generated: the volume of extravascular extracellular space (EES) per unit volume of tissue (v_e_), the blood plasma volume per unit volume of tissue (v_p_), the volume transfer constant between blood plasma and EES (K^trans^), and the rate constant between EES and blood plasma (k_ep_). While analyzing each tumor, a patient's recent (within 2 weeks) hematocrit and the relaxivity of the administered GBCA (3.8 mM^−1^ s^−1^ for meglumine gadopentetate and 3.4 mM^−1^ s^−1^ for meglumine gadoterate)^16^ were input. The arterial input function was automatically obtained.

The DSC perfusion images were analyzed using a commercially available software (IB Neuro 2.0RC; Imaging Biometrics, Elm Grove, WI). Contrast leakage-corrected maps of cerebral blood volume relative to the normal-appearing white matter (rCBV) were automatically obtained.

### Region-of-interest analysis

For each patient, maps of APTSI, ADC, pharmacokinetic parameters, and rCBV were co-registered to post-contrast T1WIs with affine transformation using the TurboReg algorithm^17^.

**Figure 1:**
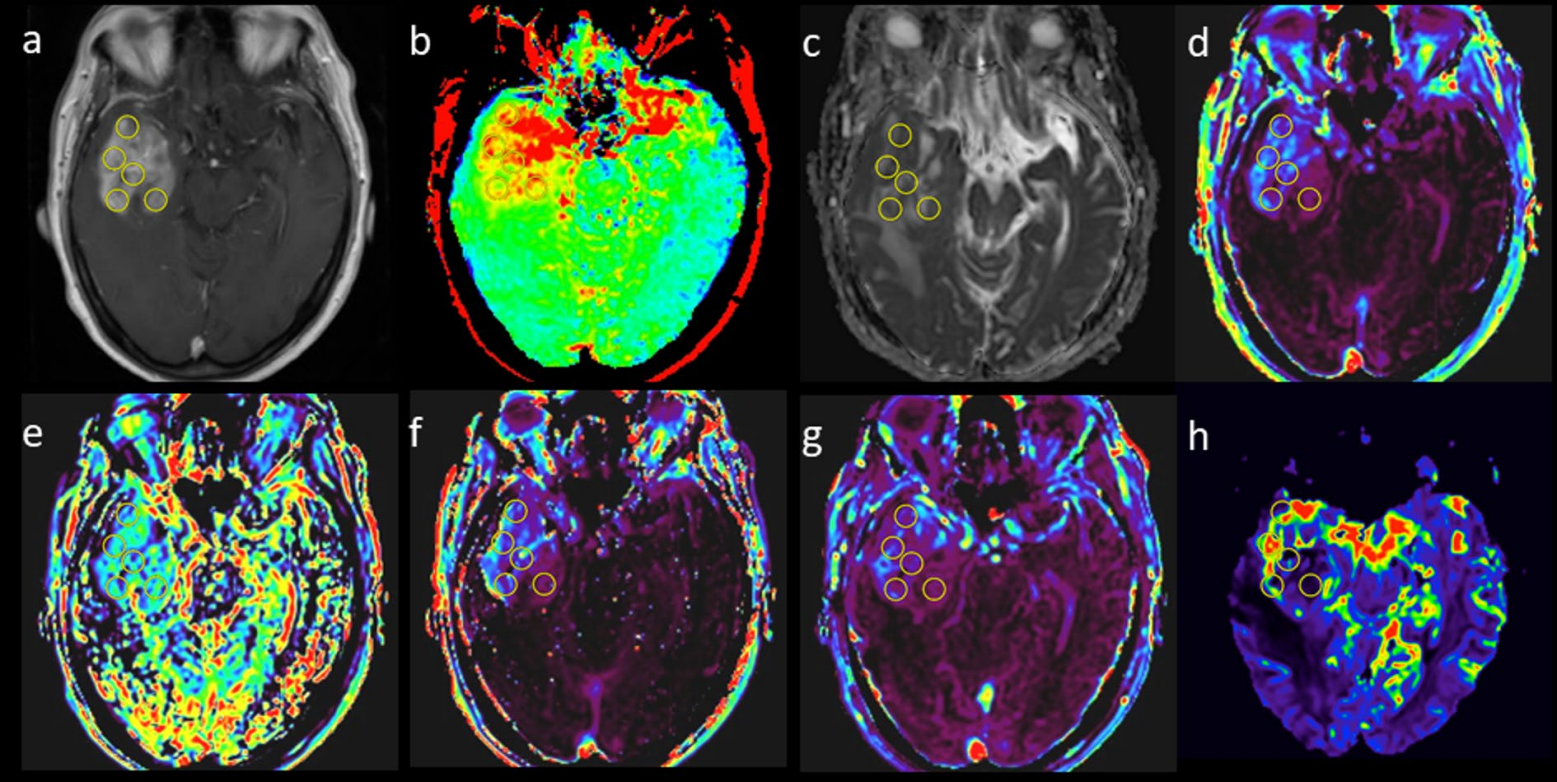
Region-of-interest (ROI) measurements in a 68-year-old female with glioblastoma. Round ROIs with a diameter of 10 mm are placed to fill the solid enhancing part of the tumor on contrast-enhanced T1-weigted image (**a**). The ROIs were copied onto maps of APTSI (**b**), ADC (**c**), K^trans^ (**d**), k_ep_ (**e**), v_e_ (**f**), v_p_ (**g**), and rCBV (**h**).

Region-of-interest (ROI) analysis was performed by two independent observers with six (observer 1) and three (observer 2) years of experience in neuroradiology. The observers placed round ROIs with a diameter of 10 mm to fill the enhancing solid part of each tumor (Fig. 1, ROIs for the other 21 patients are shown in Supplementary Fig. S1). They placed as many ROIs as possible but were careful to avoid overlaps of ROIs and exclude non-enhancing parts. ROIs were first placed on the post-contrast T1WIs and then copy-and-pasted onto the co-registered parametric maps. The mean value of each parameter was obtained for each ROI. We used relatively large ROIs rather than voxel-by-voxel comparison so that the comparisons between the parameters would be immune to spatial co-registration imperfections.

### Statistical analysis

Correlations between the quantitative MR image parameters (APTSI, ADC, K^trans^, k_ep_, v_e_, v_p_, and rCBV) measured in the same ROIs were evaluated using Spearman's rank-correlation test. The contributions of ADC, K^trans^, k_ep_, and rCBV to APTSI were analyzed using multiple regression analysis. The v_e_ and v_p_ were not included in the independent variables because of their strong correlations with K^trans^ and k_ep_ and rCBV, respectively. *P* values of < 0.05 were used to denote statistical significance. All statistical analyses were performed using Statistical Package for the Social Sciences (version 24.0; IBM Corporation, Armonk, NY, USA).

## Results

Thirty-two patients met the inclusion criteria. Three patients with recurrent tumors were excluded. Another seven patients were excluded due to artifacts. As a result, 22 patients (12 males and 10 females; age range, 42–83 years; median age, 66 years) with 22 tumors (21 glioblastomas [IDH-wildtype] and 1 anaplastic oligodendroglioma [IDH-mutant and 1p/19q-codeleted]) were finally included in the analysis.

Observers 1 and 2 placed 77 and 76 ROIs, respectively. Table 1 shows the mean and standard deviation values of each parameter obtained by the two observers.

**Table 1:** Mean values of quantitative MR imaging parameters.

	n	APTSI (%)	ADC (× 10^–3^ mm^2^/s)	rCBV	K^trans^ (× 10^–3^ /min)	k_ep_ (× 10^–3^ /min)	v_e_ (× 10^–3^)	v_p_ (× 10^–3^)
Observer 1	77	3.01 ± 0.87	1.09 ± 0.24	3.92 ± 2.33	40.00 ± 18.23	238.23 ± 125.69	246.60 ± 223.17	13.72 ± 8.85
Observer 2	76	2.96 ± 0.88	1.07 ± 0.22	3.81 ± 2.22	42.49 ± 28.61	250.94 ± 130.86	235.89 ± 205.66	14.63 ± 12.12

### Correlations between diffusion and perfusion parameters and APTSI

**Figure 2:**
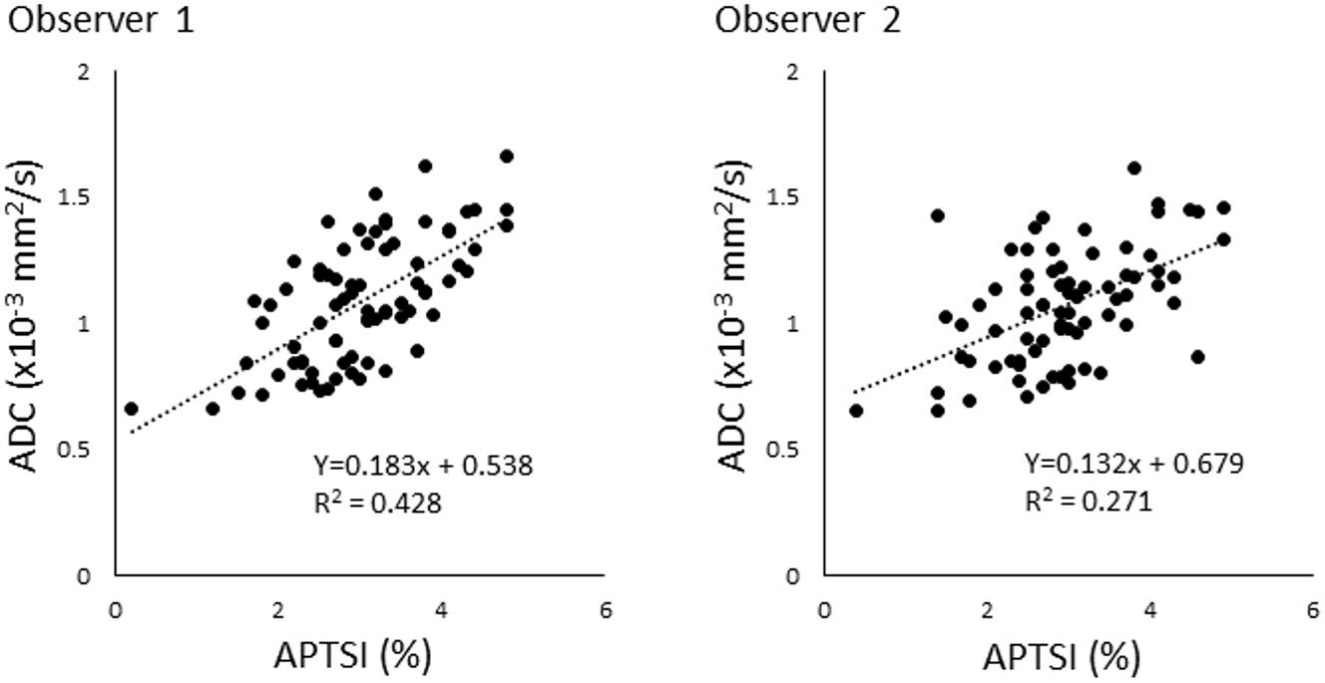
Scatter plots showing the relationships between APTSI and ADC for observers 1 and 2.

Table 2 summarizes the results of the correlation analysis. The two observers showed highly concordant results. For both observers, moderate positive correlations were observed between APTSI and ADC (ρ = 0.625 and ρ = 0.490 for observers 1 and 2, respectively; *p* < 0.001 for both). In addition, weak to moderate negative correlations were found between APTSI and k_ep_ (ρ = − 0.454 and ρ = − 0.397 and *p* < 0.001 for both) and between APTSI and v_e_ (ρ = 0.387 and ρ = 0.334 and *p* = 0.001 and *p* = 0.003 for observers 1 and 2, respectively). A weak negative correlation between APTSI and v_p_ (ρ = − 0.248; *p* = 0.029) was found only by observer 1. No other parameters showed significant correlations with APTSI (*p* > 0.05). Multiple regression analysis (Table 3) revealed that ADC was significantly associated with APTSI (*p* < 0.001 for both observers), while k_ep_ was marginally associated with APTSI only for observer 1 (*p* = 0.047). No other parameters were significantly associated with APTSI (*p* > 0.05). Figure 2 shows relationships between APTSI and ADC for observers 1 and 2.

**Table 2:** Correlations between quantitative MR imaging parameters. In each cell, numbers in upper and lower indicate Spearman’s correlation coefficients (observer 1/observer 2) and corresponding p values (observer 1/observer 2), respectively.

	APTSI	ADC	K^trans^	k_ep_	v_e_	v_p_	rCBV
APTSI	1.000	0.625/0.490	0.139/0.099	− 0.454/− 0.397	0.387/0.334	− 0.248/− 0.201	− 0.141/− 0.168
	–	< 0.001/< 0.001	0.229/0.396	< 0.001/< 0.001	0.001/0.003	0.029/0.082	0.221/0.146
ADC		1.000	0.224/0.188	− 0.397/− 0.237	0.412/0.334	− 0.219/− 0.198	− 0.051/0.045
		–	0.050/0.104	< 0.001/0.039	< 0.001/0.003	0.056/0.086	0.658/0.700
K^trans^			1.000	− 0.211/− 0.135	0.699/0.692	0.476/0.574	0.096/0.375
			–	0.066/0.244	< 0.001/< 0.001	< 0.001/< 0.001	0.406/0.001
k_ep_				1.000	− 0.807/− 0.738	0.451/0.443	0.607/0.522
				–	< 0.001/< 0.001	< 0.001/< 0.001	< 0.001/< 0.001
v_e_					1.000	− 0.066/0.020	− 0.378/− 0.160
					–	0.570/0.866	0.001/0.167
v_p_						1.000	0.584/0.692
						–	< 0.001/< 0.001
rCBV							1.000
							–

**Table 3:** Multiple regression analysis examining associations between APTSI and diffusion/perfusion imaging parameters. Values for observer 1/observer 2 are shown for each item.

	Standardized β	B	95% CI of B	P
ADC	0.585/0.476	2.093/1.877	1.423 to 2.763/1.071 to 2.683	< 0.001/< 0.001
K^trans^	− 0.033/0.009	− 0.002/0.000	− 0.010 to 0.007/− 0.006 to 0.007	0.726/0.935
k_ep_	− 0.232/− 0.202	− 0.002/− 0.001	− 0.003 to 0.000/− 0.003 to 0.000	0.047/0.075
CBV	0.084/− 0.104	0.031/− 0.041	− 0.048 to 0.110/− 0.131 to 0.048	0.434/0.359

### Correlations between diffusion and perfusion parameters

For both observers, ADC was significantly positively correlated with v_e_ (ρ = 0.412 and ρ = 0.334 and *p* < 0.001 and *p* = 0.003 for observers 1 and 2, respectively) and significantly negatively correlated with k_ep_ (ρ = − 0.397 and ρ = − 0.237 and *p* < 0.001 and *p* = 0.039, respectively). K^trans^ was significantly positively correlated with v_e_ (ρ = 0.699 and ρ = 0.692 for observers 1 and 2, respectively, and *p* < 0.001 for both) and v_p_ (ρ = 0.476 and ρ = 0.574 for observers 1 and 2, respectively, and *p* < 0.001 for both). k_ep_ was significantly positively correlated with v_p_ (ρ = 0.451 and ρ = 0.443 for observers 1 and 2, respectively, and *p* < 0.001 for both) and rCBV (ρ = 0.607 and ρ = 0.522 for observers 1 and 2, respectively, and *p* < 0.001 for both) and significantly negatively correlated with v_e_ (ρ = − 0.807 and ρ = − 0.738 for observers 1 and 2, respectively, and *p* < 0.001 for both). Finally, v_p_ was significantly positively correlated with rCBV (ρ = 0.584 and ρ = 0.692 for observers 1 and 2, respectively, and *p* < 0.001 for both).

## Discussion

Our results revealed a significant positive correlation between APTSI and ADC in ROIs within enhancing solid components of high-grade gliomas. To the best of our knowledge, no previous study has investigated the region-wise correlations between APTSI and other quantitative MRI parameters in malignant gliomas. The positive correlation between APTSI and ADC indicates that high APTSIs tend to originate in tissues with less diffusion restriction. The ADC has been linked with cell density in tumors: a significant negative correlation between ADC and histopathological cell density has been reported in several studies^18,19^. Thus, the positive correlation between APTSI and ADC in the results of this study seems inconsistent with the hypothesis that increased APTSI in high-grade gliomas originates from increased intracellular proteins and peptides in proliferating tumor cells. Recent studies have suggested contribution of extracellular proteins to ADC in glioblastoma. Pope et al.^20^ reported overexpression of various proteins in the extracellular matrix (ECM) in glioblastomas that had higher ADC in the enhancing area than those with lower ADC. Patel et al.^21^ reported overproduction of decorin in ECM in glioblastomas with higher ADC in the enhancing area. Decorin modulates the rigidity and stiffness of the ECM by binding with various ECM macromolecules and activating specific matrix metalloproteinases^22^, which may in turn increase fluid mobility within the extracellular environment. These protein overproduction and molecular changes in ECM may increase mobile proteins and peptides in ECM that generate APT-related signal.

Mobile proteins in microscopic necrotic foci and fluid collection in microcysts can be another candidates for the concurrent increase of ADC and APTSI. Togao et al.^5^ reported that gliomas with intratumoral microscopic necrosis show higher ATPSIs than those without microscopic necrosis.

A weak to moderate positive correlation was observed between APTSI and k_ep_. We assume that this is due to the confounding effect of the moderate positive correlation between ADC and v_e_, which showed a strong negative correlation with k_ep_.

Our results do not exclude the intracellular origin of APTSI in brain tumors. Note that this study included only high-grade gliomas. Moreover, our ROIs only covered enhancing solid components. A well-documented APTSI increase from low-grade to high-grade gliomas may be related to the increasing intracellular cytosolic protein; a cohort of mixed low-grade and high-grade gliomas has shown a significant positive correlation between APTSI and cell density^5,6^. We are aware that the contribution of extracellular proteins and peptides should be evaluated with caution. Extracellular pH tends to be acidic due to lactate removal from the cell, which could result in lowered APTSI through reduction of amide proton exchange rate^23^. Thus, the contribution of extracellular proteins and peptides may not be large. In addition, our results do not reject the use of APTSI as an imaging marker for malignancy and the viability of tumors.

We did not find a significant correlation between APTSI and rCBV. The blood is rich in proteins and peptides, and studies have shown that intravascular blood can generate high APTSIs^24,25^. High-grade gliomas have high blood volume resulting from a rich neovascularization stimulated by vascular endothelial growth factor. Nonetheless, our results suggest that the blood in a tumor is not a dominant factor contributing to APTSI in high-grade gliomas.

The significant positive correlations between K^trans^ and v_e_ and between K^trans^ and v_p_ found in this study conform to the findings of a study by Mills et al.^26^ and may represent a concurrent progression of tumor vascularization and permeability and the development of necrosis with tumor growth^27^. The significant positive correlation between rCBV and v_p_, which is the blood plasma volume per unit volume of tissue, was predictable.

This study has several limitations. First, this is a retrospective study involving only 22 tumors from a single institution, which may have resulted in unknown biases. A prospective study with a larger sample size is needed to confirm the findings of this study. Second, our study population consisted predominantly of glioblastomas, IDH-wildtype. Although glioblastoma, IDH-wildtype is the most common type of high-grade glioma, our findings may not be applicable to other types of high-grade gliomas. Third, the non-enhancing components of the tumors were excluded from the analyses. We included only enhancing components because in high-grade gliomas, distinguishing tumor invasion from vasogenic edema in the non-enhancing T2-prolonged areas surrounding the enhancing tumor is often difficult. Fourth, APT imaging was performed using a single-slice sequence, which limited the volume coverage. Finally, the two observers freely placed the ROIs using post-contrast images as guidance. Thus, the inter-observer reproducibility of their results could not be quantified using a statistical index. Nonetheless, their results were in close agreement.

## Conclusion

The APTSI in the enhancing component was positively correlated with ADC in high-grade gliomas. Extracellular proteins and peptides may be contributors to high APTSI in high-grade gliomas. Further studies are needed to elucidate their roles in APTSI in high-grade gliomas.

## Supplementary Information

Supplementary Figure 1.

## Data Availability

The datasets generated and/or analyzed during this study are available from the corresponding author upon reasonable request.
