# Supplementary material for: Correlation between amide proton transfer-related signal intensity and diffusion and perfusion magnetic resonance imaging parameters in high-grade glioma
Source: Sci Rep. 2021 May 27;11:11223. doi: 10.1038/s41598-021-90841-z (PMC8159950; doi:10.1038/s41598-021-90841-z)
Supplement: Supplementary file 1 — Supplementary Figure 1. [file 41598_2021_90841_MOESM1_ESM.pdf]

Correlation between amide proton transfer-related signal intensity and diffusion and perfusion magnetic resonance imaging parameters in high-grade glioma

Masanori Nakajo, MD, PhD, Manisha Bohara, MD, PhD, Kiyohisa Kamimura, MD, PhD, Nayuta Higa, MD, PhD, Takashi Yoshiura, MD, PhD

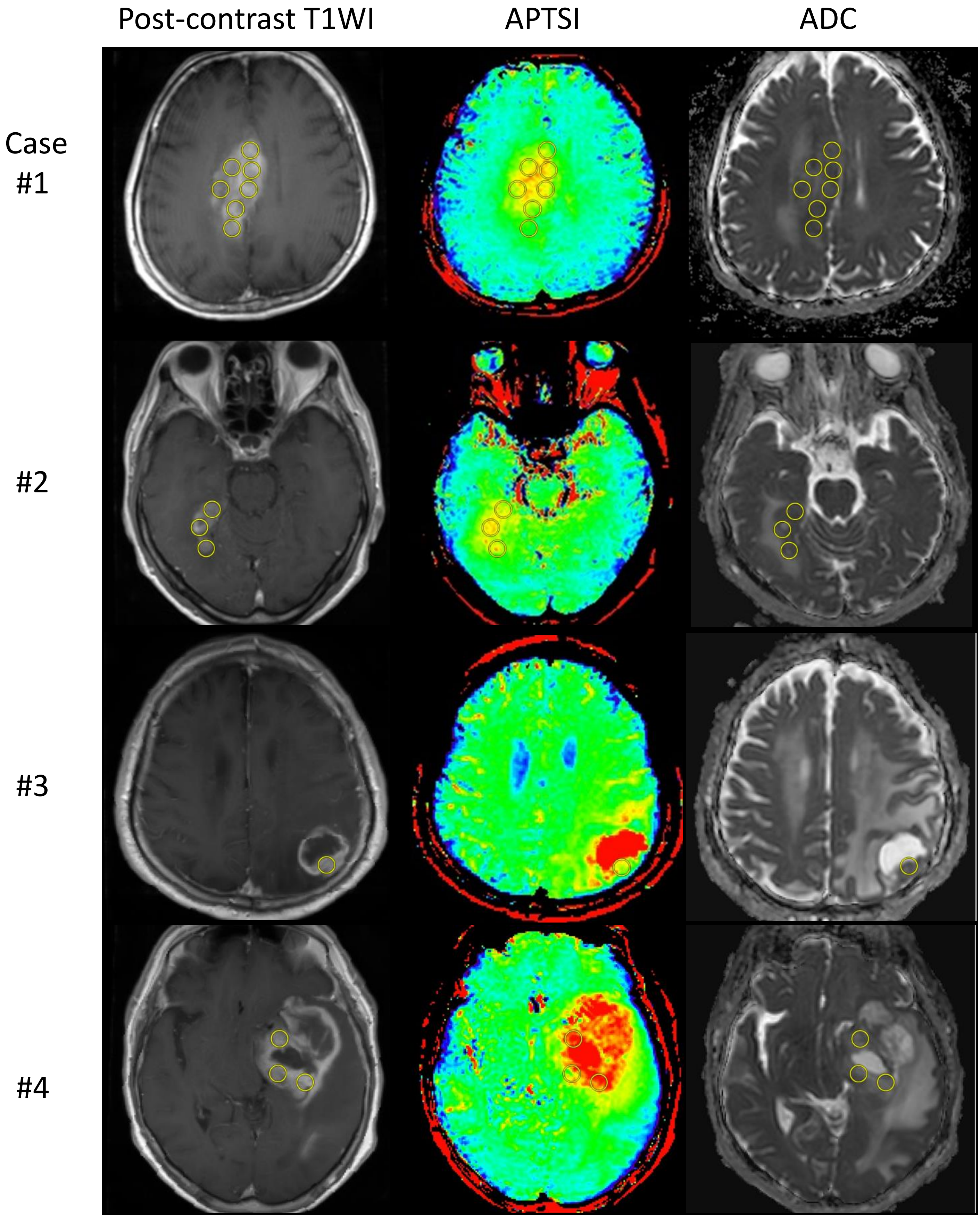
Post-contrast T1WI
APTSI
ADC
Case #1
#2
#3
#4

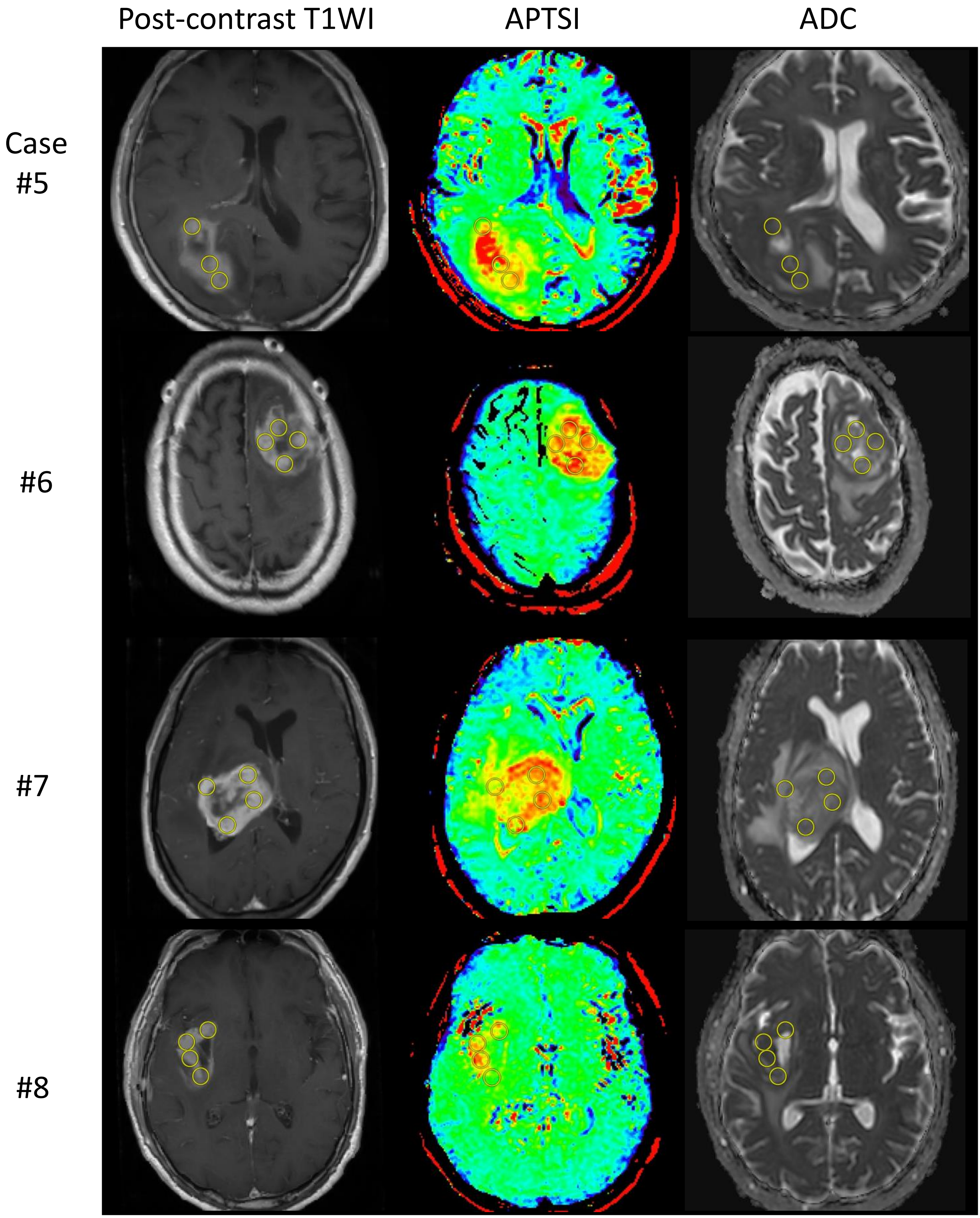

Post-contrast T1WI
APTSI
ADC
Case #5
#6
#7
#8

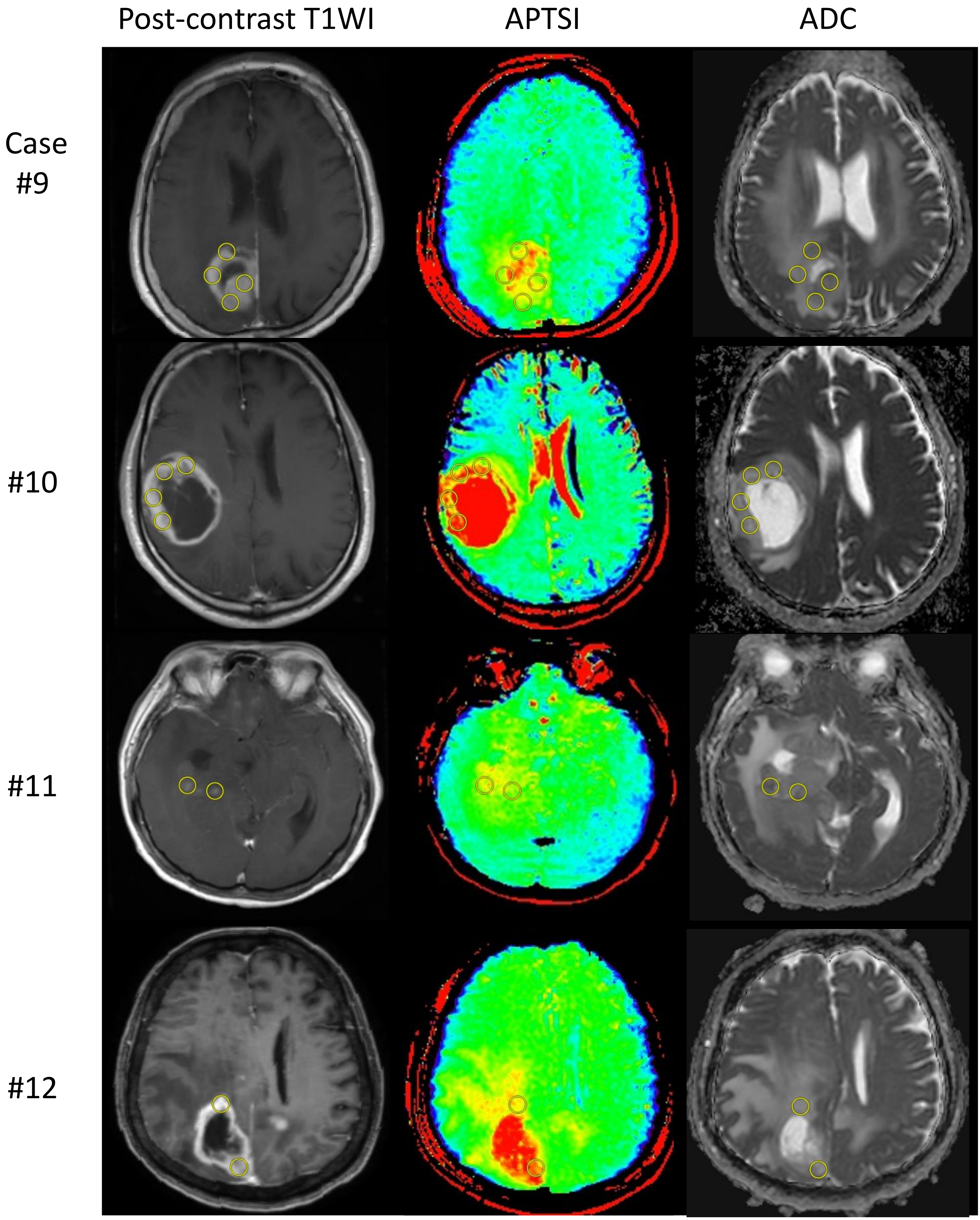
Post-contrast T1WI
APTSI
ADC
Case #9
#10
#11
#12

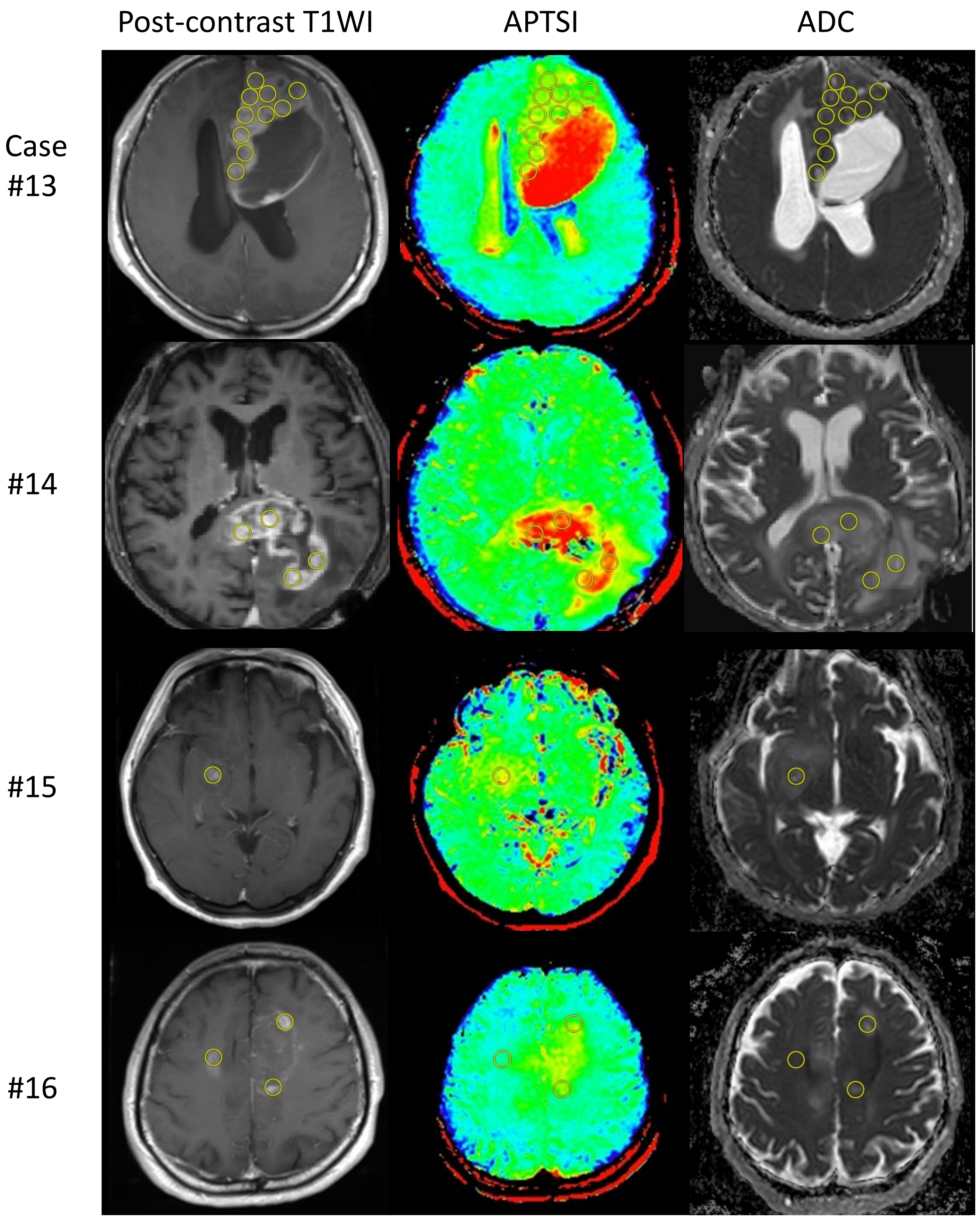
Post-contrast T1WI
APTSI
ADC
Case
#13
#14
#15
#16

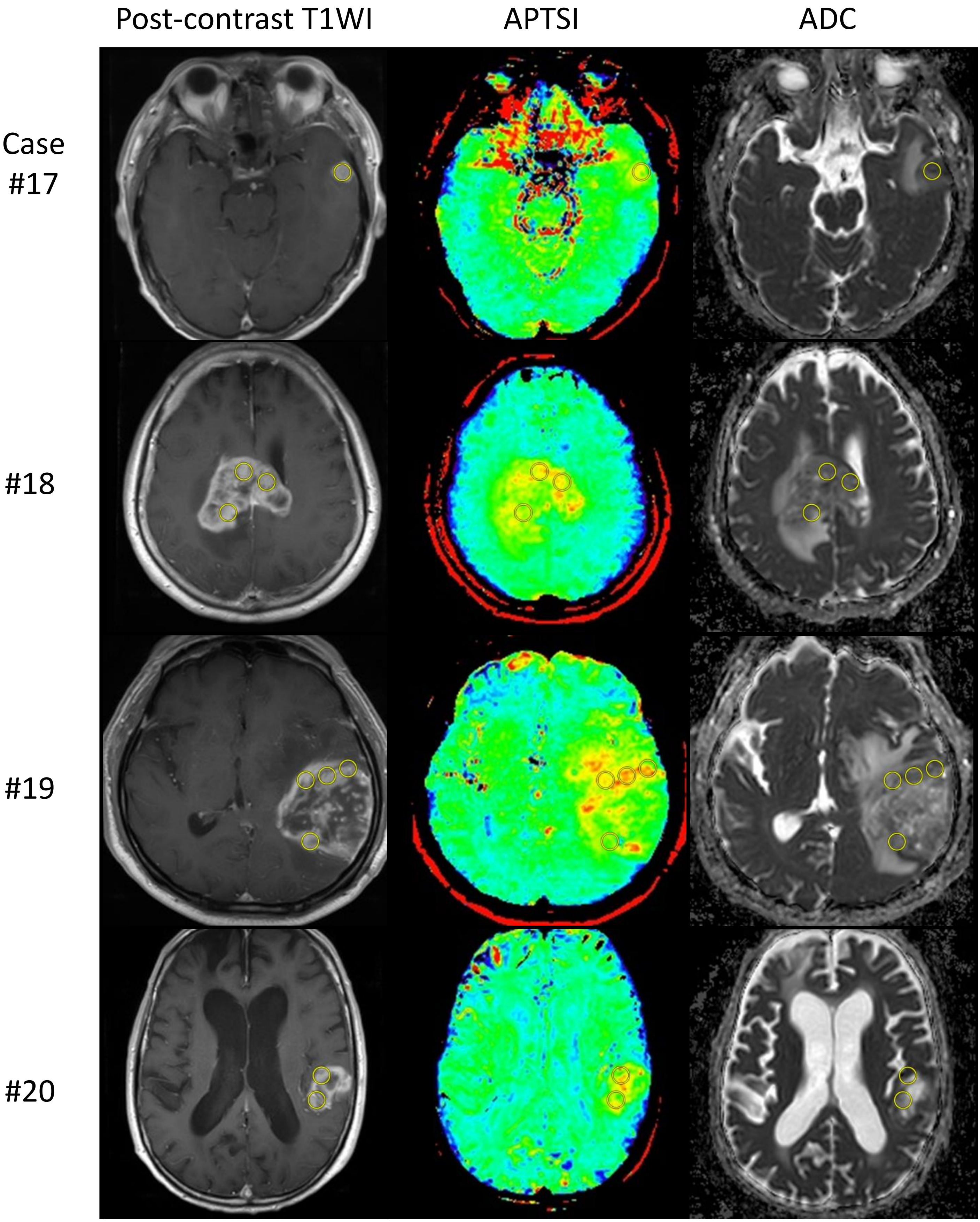
Post-contrast T1WI
APTSI
ADC
Case #17
#18
#19
#20

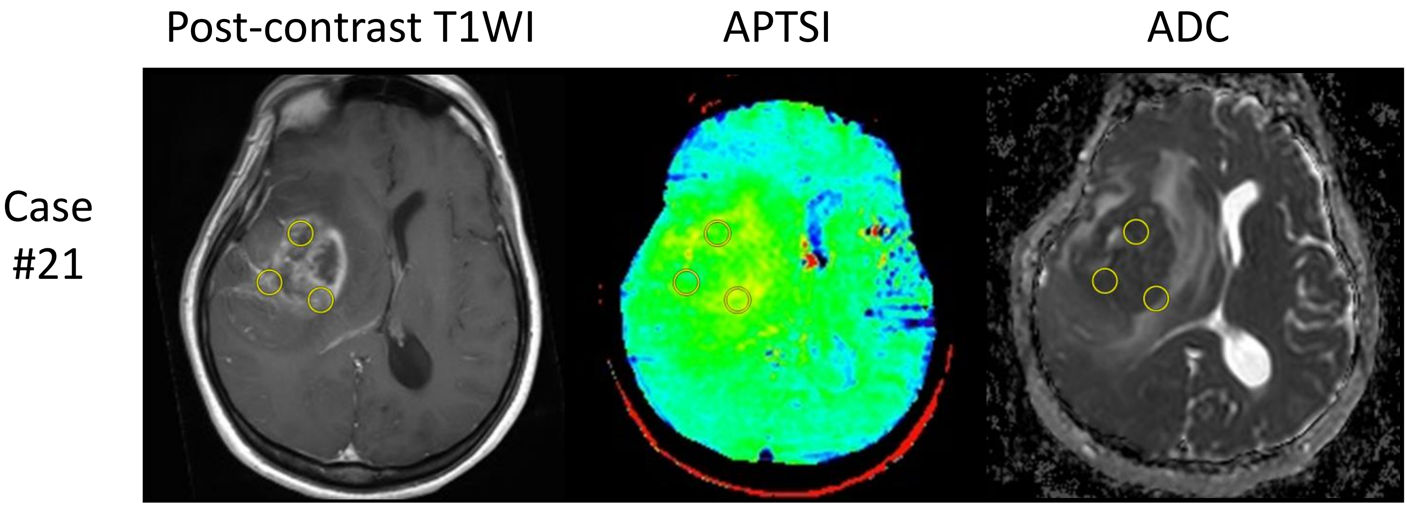

Supplementary Figure S1. ROIs on the post-contrast T1WI and maps of APTSI and ADC placed by observer 1 for all patients except the one shown in Fig. 1.
